# Causes of and Solutions to Mitochondrial Disorders: A Literature Review

**DOI:** 10.3390/ijms26146645

**Published:** 2025-07-11

**Authors:** Vera Belousova, Irina Ignatko, Irina Bogomazova, Elena Sosnova, Svetlana Pesegova, Anastasia Samusevich, Evdokiya Zarova, Madina Kardanova, Oxana Skorobogatova, Anna Maltseva

**Affiliations:** N.V.Sklifosofsky Institute of Clinical Medicine, I.M. Sechenov First Moscow State Medical University (Sechenov University), 119991 Moscow, Russiabogomazova_i_m@staff.sechenov.ru (I.B.); pesegova_s_v@staff.sechenov.ru (S.P.); kardanova_m_a@staff.sechenov.ru (M.K.); aisha27_sum@mail.ru (O.S.); maltseva_a_g@staff.sechenov.ru (A.M.)

**Keywords:** mitochondrial disorders, mitochondrial DNA, mitochondrial replacement therapy, three-parent baby, mitochondrial transplantation therapy, review

## Abstract

Mitochondria are currently of great interest to scientists. The role of mitochondrial DNA (mtDNA) mutations has been proven in the genesis of more than 200 pathologies, which are called mitochondrial disorders. Therefore, the study of mitochondria and mitochondrial DNA is of great interest not only for understanding cell biology but also for the treatment and prevention of many mitochondria-related pathologies. There are two main trends of mitochondrial therapy: mitochondrial replacement therapy (MRT) and mitochondrial transplantation therapy (MTT). Also, there are two main categories of MRT based on the source of mitochondria. The heterologous approach includes the following methods: pronuclear transfer technique (PNT), maternal spindle transfer (MST), Polar body genome transfer (PBT) and germinal vesicle transfer (GVT). An alternative approach is the autologous method. One promising autologous technique was the autologous germline mitochondrial energy transfer (AUGMENT), which involved isolating oogonial precursor cells from the patient, extracting their mitochondria, and then injecting them during ICSI. Transmission of defective mtDNA to the next generation can also be prevented by using these approaches. The development of a healthy child, free from genetic disorders, and the prevention of the occurrence of lethal mitochondrial disorders are the main tasks of this method. However, a number of moral, social, and cultural objections have restricted its exploration, since humanity first encountered the appearance of a three-parent baby. Therefore, this review summarizes the causes of mitochondrial diseases, the various methods involved in MRT and the results of their application. In addition, a new technology, mitochondrial transplantation therapy (MTT), is currently being actively studied. MTT is an innovative approach that involves the introduction of healthy mitochondria into damaged tissues, leading to the replacement of defective mitochondria and the restoration of their function. This technology is being actively studied in animals, but there are also reports of its use in humans. A bibliographic review in PubMed and Web of Science databases and a search for relevant clinical trials and news articles were performed. A total of 81 publications were selected for analysis. Methods of MRT procedures were reviewed, their risks described, and the results of their use presented. Results of animal studies of the MTT procedure and attempts to apply this therapy in humans were reviewed. MRT is an effective way to minimize the risk of transmission of mtDNA-related diseases, but it does not eliminate it completely. There is a need for global legal regulation of MRT. MTT is a new and promising method of treating damaged tissues by injecting the body’s own mitochondria. The considered methods are extremely good in theory, but their clinical application in humans and the success of such therapy remain a question for further study.

## 1. Introduction

In 1988, two parallel discoveries were made that introduced the era of “mitochondrial disorders”. Anita Harding from the Department of Clinical Neurology, Institute of Neurology, Queen Square, London, UK, identified large-scale single deletions of mitochondrial DNA (mtDNA) in muscle biopsy specimens from patients with “mitochondrial myopathies” [1]. That same year, Douglas C. Wallace from Emory University, Atlanta, Georgia, discovered a point mutation in the mtDNA gene associated with Leber’s hereditary optic neuropathy, inherited maternally in several generations of the same family [2]. These two discoveries have sparked renewed interest in pathologies associated with mutations in the tiny mtDNA molecule. Currently, more than 200 diseases associated with mitochondrial pathology are known. The main and most severe of them are summarized in Table 1. Many patients do not show these full-blown clinical pictures and are affected by a variety of complex or partial presentations (myopathies, neuropathies, cardiomyopathies, encephalomyopathies, multisystemic diseases, etc.) [3].

And while the nature of these pathological conditions was previously unclear, nowadays about 780 children in the USA are diagnosed with mitochondria-related diseases every year [4]. In the UK, about 100 children are born with severe disorders associated with mitochondrial pathology, most of whom die in infancy [5].

The effects of mitochondrial diseases are very diverse. Due to the different distribution of defective mitochondria in different organs, a mutation in one person may result in liver disease and in another in brain disease. The clinical manifestations of mitochondrial pathology can be significantly altered, slowly building up over time. Some small defects result only in the patient’s inability to tolerate age-appropriate physical activity and are not accompanied by serious painful manifestations. Other defects may be more dangerous, leading to serious pathology.

The majority of mitochondrial diseases affect the nervous and muscular systems because the cells of these tissues have a large number of mitochondria. Mitochondrial diseases are currently incurable. Patients receive only symptomatic treatment, which usually insignificantly improves their quality of life. Therefore, the study of this pathology, as well as methods of its correction and, most importantly, attempts to prevent the transmission of mutated mitochondria from a mother to her child, are currently among the urgent and interesting tasks of modern science. Due to the rapid development of genome editing technologies, including mitochondrial genome editing, new opportunities for solving this medical problem are opening up.

## 2. Mitochondria: Structure and Function

**Figure 1:**
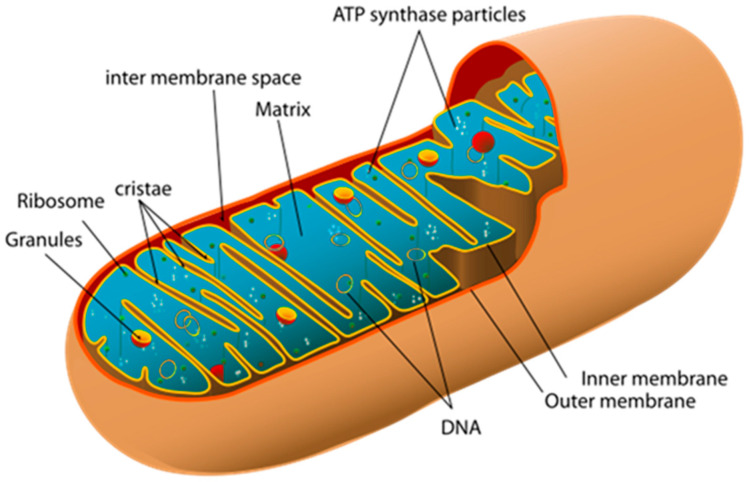
Structure of a mitochondrion. https://commons.wikimedia.org/wiki/File:Animal_mitochondrion_diagram_en.svg#/media/File:Animal_mitochondrion_diagram_en.svg (accessed on 3 December 2012).

A mitochondrion is a semi-autonomous, DNA-containing, double-membrane organelle of a eukaryotic cell. They were discovered by Albert von Kölliker in 1857 in the voluntary muscles of insects. Mitochondria consist of an outer and inner membrane, an intermembrane space, and a matrix. The inner membrane forms folds—cristae—that increase the inner surface. The outer membrane contains porin, a channel-forming protein that forms openings through which small molecules and ions can pass, which plays an important role in the transportation of lipids and calcium ions. The enzyme complexes of cellular metabolism, which carry out electron transport and ATP synthesis, are located on the cristae. In the intermembrane space, there are enzymes of antioxidant defense (catalase, superoxide dismutase, glutathione peroxidase), various modulators of cell death, such as cytochrome C, apoptosis-inducing factor (AIF), and endonuclease G, which causes chromatin fragmentation independently of caspases, etc., in the matrix. The matrix contains enzymes of the Krebs cycle, urea cycle, protein-synthesizing apparatus and mitochondrial DNA (mtDNA), 70S ribosomes (Figure 1) [6].

Traditionally, mitochondria are described as “energy factories of the cell”, where ATP is synthesized by oxidation of organic substrates. The role of mitochondria in the regulation of calcium homeostasis, cellular metabolism, proliferation, and apoptosis in somatic and germ cells is not insignificant. They are essential for the metabolism of cholesterol, neurotransmitters, estrogens, and testosterone, and they contain enzymes for the formation of pyrimidines and heme.

Mitochondria, via the respiratory chain, are involved in the production of reactive oxygen species (ROS). In turn, excessive ROS can lead to oxidative stress (OS), which can cause gene mutations, protein denaturation, and lipid peroxidation directly or indirectly. This is reflected in decreased cellular ATP levels, increased cytoplasmic Ca2+ levels, inflammation, and so on. Consequently, ROS are considered to be significant risk factors for human aging and the development of various diseases, including diabetes, cardiovascular and neurodegenerative diseases [7].

The number of mitochondria varies in different human cells, and this is directly related to how much energy the cell needs to function. For example, in human skin cells there are on average 5–6 mitochondria, in muscle cells—up to 1000, in liver cells—up to 2500 [8]. The record holders in the number of mitochondria are oocytes, each of which may contain from 100,000 to 600,000 mitochondria, whereas in a spermatozoon there are only 100. Mitochondria are inherited by embryos only through the maternal line, as sperm mitochondria are destroyed during fertilization [9].

High energy-consuming organs and tissues, such as the central nervous system, heart, visual organs, and skeletal muscle, are particularly susceptible to energy deficiency due to defects in oxidative phosphorylation. The central nervous system (CNS) is the most vulnerable to mitochondrial dysfunction due to its dependence on aerobic metabolism and oxidative phosphorylation. The inability of neurons to renew themselves effectively leads to central and peripheral nervous system dysfunction, which contributes to the development of neurodegenerative diseases (Alzheimer’s disease, Parkinson’s disease, Huntington’s disease, and amyotrophic lateral sclerosis) manifested by motor impairment, cognitive decline, and memory impairment. Although the pathologic changes in different brain regions and clinical symptoms in neurodegenerative diseases differ, common features can be observed at the genetic, cellular, or molecular level, including changes in cellular morphology and mitochondrial dysfunction. Mitochondrial density in neurons is lower than in muscle cells, for example, but the brain consumes almost ten times more oxygen and glucose than other tissues [10].

## 3. Mitochondrial DNA

Mitochondria are the only cell organelles that have their own so-called mitochondrial DNA (mtDNA), which consists of two chains: L (light) and H (heavy). The mtDNA genome includes 37 structural genes, of which 13 encode subunits of oxidative phosphorylation complexes, 22 encode transport RNA, and 2 encode the large subunit of ribosomes [11]. In contrast to nuclear DNA, mtDNA is 10–20 times more likely to be mutated and less efficiently repaired because it is not protected by histones and, in addition, is located in close proximity to the source of reactive oxygen species.

The first mutations in mitochondrial DNA were discovered in 1988, and since then more than 200 mutations have been identified [12]. Each cell contains hundreds or thousands of mitochondria, and each mitochondrion contains multiple copies of mtDNA. This phenomenon is called polyplasmy. Normal cells have identical copies of mtDNA, which is called homoplasmy. When mutated mtDNA is present, heteroplasmy is observed [13]. Generally, a cell is insensitive to the presence of mitochondria with mutant DNA until their number reaches a certain threshold of 50–70%. It is mtDNA heteroplasmy that causes mitochondrial diseases and also contributes to the development of such diseases as diabetes, cancer, Parkinson’s, and Alzheimer’s diseases [9,14].

Mitochondrial dysfunction can be caused, among other things, by viral diseases. For example, the COVID-19 pandemic caused by the SARS-CoV-2 virus introduced the medical community to the phenomenon of the so-called “postviral syndrome following SARS-CoV-2 infection”—a condition characterized by the development of persistent symptoms after the acute phase of infection, among which chronic fatigue, cognitive impairment, and exercise intolerance predominate, indicating systemic changes beyond the initial viral pathology [15].

Since mitochondrial inheritance occurs only through the maternal line, it is possible to prevent the transmission of mutated mtDNA from mother to embryo if mitochondrial pathology is detected in the family. In this case, preimplantation genetic diagnosis is recommended. The test is performed on one or more cells taken from an early embryo, which allows selection of embryos with a low percentage of mutations for subsequent transfer into the uterus [9]. However, it is not uncommon to detect homoplasmy (when all mtDNA contains mutations) or high levels of heteroplasmy, and then there are great difficulties in selecting embryos with a lower mutation load [16].

## 4. Diagnostics of Mitochondrial Disorders

Given the genetic and phenotypic heterogeneity of mitochondrial genetic disorders, accurate diagnosis of these diseases remains challenging. Clinical symptoms can range from nonspecific fatigue, exercise intolerance, and weakness to syndromal phenotypes [17]. Mitochondrial disease phenotypes are often manifested by developmental delay, the appearance of seizures, hypotonia (myopathies), and visual impairment (retinopathies). For example, one of the first signs of primary mitochondrial disease may be epilepsy, which is quite difficult to treat and often has a poor prognosis [18]. In addition to careful and detailed clinical observation, comprehensive testing including biochemical analysis of biological fluids, neuroimaging, and DNA and RNA sequencing is necessary. Thus, anemia or neutropenia detected by clinical blood tests may indicate some mitochondrial genetic diseases such as, for example, Pearson syndrome [19]. Cytopenia and sideroblastic anemia occur in 10–30% of patients with confirmed mitochondrial diseases and may present initially or increase over time [20].

Human induced pluripotent stem cells (iPSCs) represent a promising approach for developing human model systems and evaluating therapeutic options in the context of a specific patient and tissue. iPSCs are increasingly being used to study mitochondrial diseases, either to identify mutations in two-dimensional (2D) or three-dimensional (3D) models or to study the impact of potential treatment options [21].

## 5. Mitochondrial Therapy

**Figure 2:**
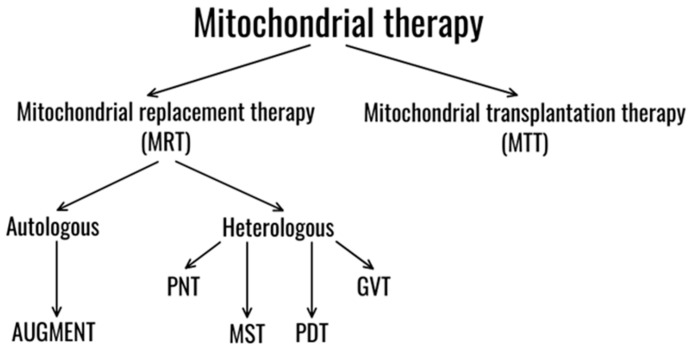
Trends of mitochondrial therapy. Created with supa.ru.

There are two main trends of mitochondrial therapy: mitochondrial replacement therapy (MRT) and mitochondrial transplantation therapy (MTT) (Figure 2). One of the most effective methods to prevent the transmission of mutated mtDNA from mother to child is currently mitochondrial replacement therapy (MRT) [22]. This method cannot be used to treat an adult, but it allows the mother, a carrier of the mutation, to give birth to a healthy child without mutations in mtDNA. The UK became the first country to pass legislation approving its use in 2015 [23].

**Figure 3:**
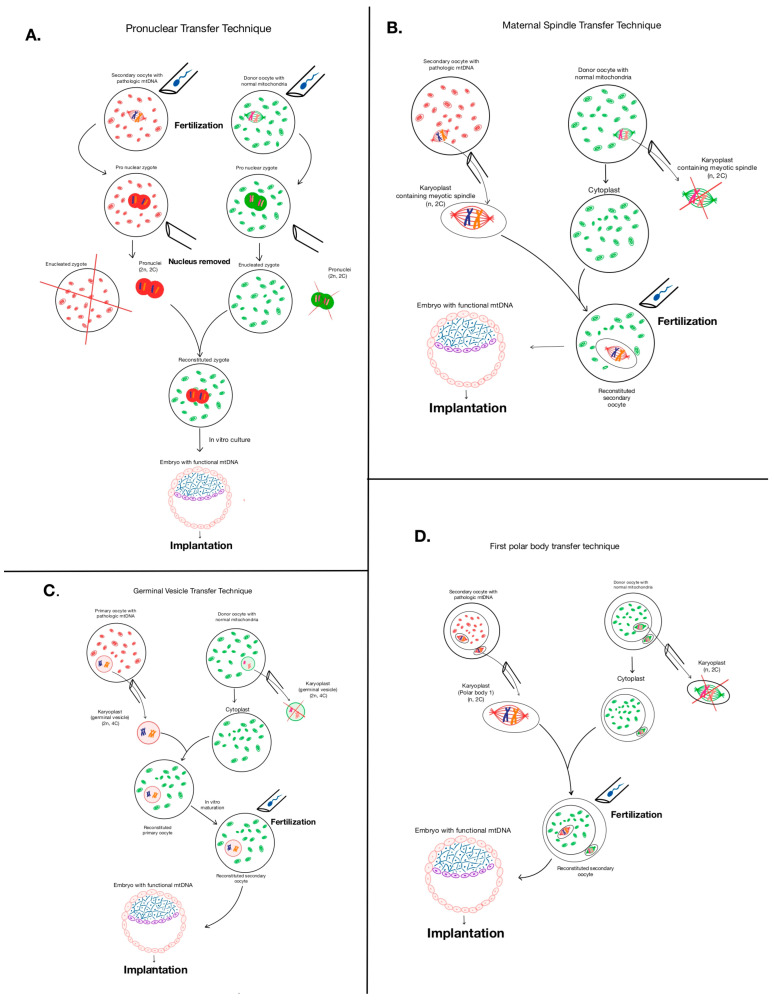
Four methods of heterologous approaches in mitochondrial replacement therapy. (**A**) Steps involved in mitochondrial donation by pronuclear transfer technique (PNT); (**B**) steps involved in mitochondrial donation by maternal spindle transfer method (MST); (**C**) steps involved in mitochondrial donation by germinal vesicle transfer (GVT); and (**D**) steps involved in mitochondrial donation by polar body genome transfer (PBT).

Additionally, there are two main categories of MRT based on the source of mitochondria. The heterologous approach includes the following methods: pronuclear transfer technique (PNT), maternal spindle transfer (MST), polar body genome transfer (PBT), and germinal vesicle transfer (GVT) (Figure 3) [24].

An alternative approach is the autologous method. One promising autologous technique is the autologous germline mitochondrial energy transfer (AUGMENT), which involves isolating oogonial precursor cells from the patient, extracting their mitochondria, and then injecting them during ICSI [25]. Each of these directions will be discussed further in our review.

### 5.1. Mitochondrial Replacement Therapy—The Heterologous Approach

#### 5.1.1. Pronuclear Transfer Method

The essence of the method is that in vitro donor and mother oocytes are fertilized by paternal spermatozoa. One of the zygotes belongs to the biological parents with pronuclei and defective mitochondria, and the other belongs to the donor with pronuclei and healthy mitochondria. The pronuclei of the biological parents are extracted and transplanted into the donor zygote (with rejected pronuclei) with healthy mitochondria. A blastocyst is formed, which is then examined for mitochondrial mutations before implantation into the uterus. As a result, the child receives DNA from three people: nuclear DNA from the mother and father, and mitochondrial DNA from the donor (Figure 3A) [26].

#### 5.1.2. Maternal Spindle Transfer Method (MST)

The essence of this method of mitochondrial replacement therapy is that a maternal spindle complex is extracted from a maternal oocyte with mutated mtDNA at the metaphase stage. This complex is then transplanted into the perivitelline space of a donor oocyte with healthy mitochondria (Figure 3B) [27]. The transformed embryo is then transferred into the mother’s uterus. This approach is preferable because the maternal spindle contains little cytoplasm, which ultimately reduces the likelihood of transferring mutated mtDNA [28].

#### 5.1.3. Polar Body Genome Transfer (PBT)

Regarding this method, it is worth recalling that the polar body is formed in the process of oogenesis as a result of the first and second meiotic divisions. The polar body is a small cell with a small amount of cytoplasm and, consequently, a small number of mitochondria, which minimizes the risk of transmission of mutated mtDNA. The second division of the secondary oocyte produces one haploid oocyte and a second polar calf. The first polar calf sometimes also divides into two small cells. These transformations of the primary oocyte result in one oocyte and three polar bodies [29]. The idea of using polar bodies was first proposed by Wakayama and Yanagimachi and later adopted by Wang et al. (2014) to perform the technique in mice where, the transfer of first and the second polar body led to the normal progression of the progeny [30]. The polar body was transferred into the oocyte during the division stage and after removal of the nucleus from the oocyte. The reconstructed oocytes were subjected to intracytoplasmic sperm injection (ICSI) and cultured to blastocysts (Figure 3D) [30].

#### 5.1.4. Germinal Vesicle Transfer (GVT)

The germinal vesicle is a large nucleus present in immature (primary) oocytes. Primary oocytes with the nucleus as a germinal vesicle remain in the ovary for a long time, arrested in prophase 1. Once such an oocyte begins its maturation in the next menstrual cycle. The essence of the GVT method is to transplant the germinal vesicle from a patient’s primary oocyte into a donor oocyte from which the germinal vesicle was also extracted (Figure 3C). Since these are immature oocytes, they undergo an in vitro maturation process to complete the meiosis. After that, such a donor oocyte with the maternal germinal vesicle is fertilized with the father’s sperm and implanted [31].

With all the logic and harmony of the theory, this method has proven to be extremely ineffective. This is probably due to the fact that the techniques for in vitro maturation available today reduce the potential development of oocytes. Given the poor results obtained in animal models, it is assumed that this technique will not be employed in human oocytes until the in vitro maturation procedures are optimized [32].

### 5.2. Risks Associated with Mitochondrial Replacement Therapies (The Heterologous Approach)

Currently, the following groups of risks (problems) associated with MRT are identified in the following document: Risks derived from micromanipulation, mitochondrial carry-over, and mito-nuclear incompatibility [32].

Risks derived from micromanipulation are risks associated with the oocyte handling within the laboratory, in particular cytoskeletal disruptors, nuclear genome damage, and integration of the Sendai virus genome, which is employed to fuse the karyoplast with the cytoplast, into the embryo genome.

Mitochondrial carry-over is the transfer of maternal mtDNA together with nuclear DNA into a donor oocyte. However, MRT methods such as the polar body genome transfer allows for the minimization of this type of complications.

Despite the existence of mtDNA, many proteins that play an important role in mitochondrial functions are encoded by nuclear DNA. Thus, proper mitochondrial function (electron transfer chain, mtDNA replication, etc.) depends on the coordination of proteins encoded by nuclear and mitochondrial DNA [33]. In MRT, the mechanism of inheritance of all DNA (nuclear and mitochondrial) from one parent (the mother) is disrupted. Maternal nuclear DNA is found in a foreign environment together with mitochondrial DNA, which is alien to itself. Of course, in such a situation, there is a risk of incompatibility of two different DNAs (mito-nuclear incompatibility) in one cell.

Due to the risks described in 2015 FDA requested that the Institute of Medicine (IOM) produce a consensus report regarding the ethical and social policy issues related to genetic modification of eggs and zygotes to prevent transmission of mitochondrial disease. The report concluded that MRT in humans is ethically permissible as long as certain conditions and principles are met. One condition is that treatment should be limited to women who are at risk of transmitting severe mitochondrial disease, and because female embryos would result in heritable genetic modification, MRT research should be restricted initially to male embryos [34].

### 5.3. Results of Mitochondrial Replacement Therapy (The Heterologous Approach)

The first baby born using the mitochondrial donation method was born on 6 April 2016 in Mexico. The procedure was successfully performed by Dr. John Zhang and his team [35]. The patient was a 36-year-old Jordanian national who was a carrier of a genetic mitochondrial mutation (Leigh Syndrome), which caused four spontaneous miscarriages and the death of the patient’s two children in infancy. In this case, the team led by Dr. Zhang removed a nucleus from a healthy donor oocyte and replaced it with a nucleus taken from the woman’s oocytes, leaving the donor’s healthy mitochondria intact. The scientists then fertilized the modified egg with the father’s sperm before implanting it in the mother’s uterus. Using this method, the resulting embryos contained >99% donor mtDNA. Donor mtDNA was stably maintained in embryonic stem cells (ES cells) derived from most embryos. However, some cell lines demonstrated a gradual loss of donor mtDNA and reversion to the maternal haplotype. Unfortunately for the scientific world, the child’s parents refused any further mitochondrial testing of their child unless medically necessary [36].

It is likely that this reversion of mtDNA to maternal abnormalities is due to the fact that mitochondrial functionality and its regulation are strictly controlled by a balanced interaction between nuclear and mitochondrial DNA. Epigenetic markers such as methylation, hydroxymethylation, and miRNAs encoded by nuclear DNA have been found in mitochondria. They are imported into mitochondria and regulate the dynamics of mitochondrial genome gene expression [37].

The world’s second three-parent baby was conceived by a 34-year-old woman on 5 January 2017 in Ukraine. The pronuclear transfer method was successfully applied by Dr. Valeriy Zukin at the Nadezhda Clinic in Kiev. Reports also confirm that seven children have been born at the Nadia Clinic so far, the most recent being a boy born on 18 December 2018 [38]. Unfortunately, we did not find any information about the health status of these children anywhere. Probably, their parents, as well as the patient of Dr. John Zhang, refused further publicity.

### 5.4. Mitochondrial Replacement Therapy—The Autologous Approach

Autologous germline mitochondrial energy transfer is the transfer of autologous mitochondria isolated from oocyte precursor or oogonial stem cells (OSCs) into her oocyte together with a sperm by intracytoplasmic sperm injection (ICSI) [39].

Scientists have long known about the presence of adult stem cells in organs and tissues. These stem cells are used to repair the organ in question in the event of damage. Stem cells have been found in almost all organs, including bone marrow, skin, gastrointestinal tract, blood, lungs, skeletal muscles, heart muscle, brain, testicles, and ovaries. To avoid abnormal tissue growth, adult stem cells are found in relatively low abundance and are generally maintained in a quiescent state until the need for their activity triggers their division [40].

In 2004 J. Johnson first published a report on the existence of a self-renewing population of oocyte-generating progenitor cells termed female germline stem cells or oogonial stem cells (OSCs) [41]. Five years later, OSCs were isolated from mouse ovaries, expanded, and characterized ex vivo. Importantly, it has been demonstrated that OSCs are able to generate fertilization-competent oocytes, giving rise to viable offspring [42].

These studies involved mouse and rat oocytes, followed by studies confirming the oocyte-forming properties of OSCs isolated from human ovaries [43,44].

Meanwhile, electron microscopy studies have shown that mitochondria present in human OSCs are ultrastructurally indistinguishable from mitochondria found in human oocytes, while both mitochondrial populations differ significantly in appearance from mitochondria present in somatic cells [45].

**Figure 4:**
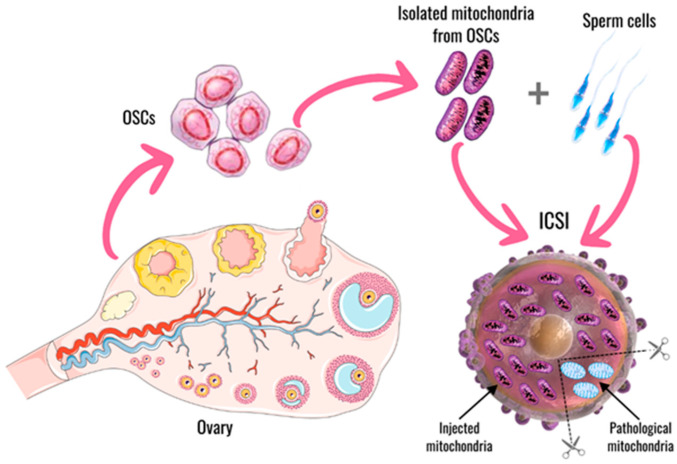
Steps involved in mitochondrial donation by autologous germline mitochondrial energy transfer technique. Created with supa.ru.

The AUGMENT procedure is as follows: autologous mitochondria from the patient’s own OSCs are isolated. Then, these mitochondria with sperm cells are injected into the poor-quality oocyte at the time of ICSI. This bolus of pristine mitochondria provides an otherwise compromised oocyte with sufficient energetic potential for successful fertilization and subsequent embryonic development, restoring the natural potential to achieve a healthy pregnancy (Figure 4) [39]. This introduction of the oocyte’s own mitochondria enhances fertility, without the drawback of having “foreign” mitochondria present in the resulting embryos and offspring. In other words, there was an opportunity to improve oocyte health with a rich source of high-energy mitochondria from an autologous source of natural oocyte progenitor cells, and the concept of AUGMENT was born [39].

The article reported the successful results of this technology in 104 patients at three different clinics at three different sites with 104 total patients. Briefly, their positive results are as follows: 104 cycles of AUGMENT performed across the three international sites have already produced four live births of six babies, nearly comparable to the total of only five live births achieved in this same patient population after previously undergoing 3.5-fold more IVF (in vitro fertilization) attempts (369 prior cycles) without AUGMENT [39].

However, then came word of the results of a fourth study in Valencia, Spain. This fourth trial of AUGMENT-IVF, reported 4 years later with 57 enrolled subjects, failed to show a clinical benefit of the procedure for enhancing cumulative live birth rates versus those obtained with IVF alone. The aim of this research was to study if autologous mitochondrial transfer (AUGMENT) improves outcome in patients with previously failed in vitro fertilization (IVF). The conclusion was that AUGMENT does not seem to improve prognosis in this population. The study has been discontinued [39,46].

Since there are currently no reliably positive results of complex procedures performed to try to avoid inheritance of pathological mitochondria from mother to child, scientists have turned their attention to a new type of mitochondrial therapy—Mitochondrial transplantation therapy.

## 6. Mitochondrial Transplantation Therapy

Mitochondrial transplantation therapy (MTT) is referred to as the procedure of injecting isolated mitochondria into the damaged area of an organ.

In 2008, for the first time, James D. McCully et al. isolated mitochondria from healthy tissue of New Zealand white rabbits and injected them into the area of myocardial ischemia, which was accompanied by a decrease in the area of ischemia and improvement of biochemical indices of ischemia markers. The authors also found that the introduction of mitochondria reduced the level of oxidative damage in the lesion area [47]. Subsequently, James D. McCully et al. isolated mitochondria from skeletal muscle and injected them into the area of myocardial ischemia. The authors showed that the injected mitochondria penetrate into cardiomyocytes and normalize myocardial contractile activity in the ischemia zone [47,48].

A study by S. Shanmughapriya et al. (2020) showed that mitochondria transferred from healthy cells into damaged cells can integrate with local mitochondria, replacing damaged ones and replenishing the number of mitochondria to meet the energy needs of the cell [49]. However, the mechanism by which transplanted exogenous mitochondria are incorporated into cells under pathological conditions has not been studied yet.

A recently discovered form of intercellular transfer—in which healthy mitochondria are actively incorporated into damaged cells, contributing to the restoration of their viability, reduction of inflammatory processes, and normalization of calcium dynamics—has been named intercellular mitochondrial transfer (MT). MT can occur involving various cellular structures and mechanisms: tunneling nanotubes (TNTs), gap junctions (GJs), extracellular vesicles (EVs) and other mechanisms (cell fusion, mitochondrial extrusion and mitocytosis mediated by migrasomes) under various conditions such as physiological as well as pathological (hypoxia, inflammation and cancer). As MT mediated by mesenchymal stromal/stem cells (MSCs) has emerged in recent years as a crucial regulatory and repair mechanism for cell and tissue regeneration and damage repair, its potential in stem cell therapy has attracted increasing attention. In particular, several articles have reported the potential therapeutic role of MSCs through MT and membrane vesicle release [50]. There have been several studies of mitochondria isolated from various tissues including muscle, liver, heart, placenta and brain and it has been shown that almost any healthy tissue or cell far from the site of injury can be used as a source for mitochondrial isolation [51].

### 6.1. Sources of Mitochondria

The sources of mitochondria could be either the patient’s own tissues or donor mitochondria. To minimize transplant risks, ideally, of course, it is preferable to use the patient’s own mitochondria from the organ whose treatment is required, because mitochondria can be tissue specific. For instance, the derivation of mitochondria from the tissues of a healthy area of the liver or heart and their delivery into the damaged area of the same organ. However, the risks of intervention on a partially damaged organ should be taken into account, as this may worsen the underlying problem. In addition, in case of brain damage, this is extremely difficult. Therefore, autogenic mitochondria from other tissues (for example, liver, muscles) are used for mitochondrial transplantation into the brain at this stage of research. But allogeneic mitochondria could be isolated from any donor tissue (excluding the brain), including mesenchymal stem cells. At the same time, the possible outcomes of allogeneic transplantation should be studied [52].

G. Ramirez-Barbieri et al. (2019) demonstrated that after intraperitoneal injection of allogeneic mitochondria, there were no direct or indirect, acute or chronic negative immune responses to them [53]. And we also did not find any information about the negative effects of MTT. With the exception of the usual functions, transplanted mitochondria can have an anti-inflammatory effect [54], increasing the activity of macrophages and T-lymphocytes and stimulating the immune system [55]. This procedure can increase the number of mitochondria and improve the function of damaged mitochondria, resulting in increased adenosine triphosphate levels, decreased reactive oxygen species production, improved Ca2+ buffering capacity, modulated inflammatory response, and reduced apoptosis to protect cells, thus promoting tissue repair [52].

There is interesting evidence that female mouse-derived mitochondria show a higher protein content, pyruvate dehydrogenase activity, and ATP production capacity than male-derived mitochondria [56].

Mitochondrial transplantation is currently being actively studied in animals for Neurodegenerative and Neurovascular Disorders particularly Parkinson’s disease, Alzheimer’s disease, stroke, schizophrenia, trauma, multiple sclerosis. In spinal cord injury, unfortunately, no reliable improvement was obtained. However, in traumatic brain injury mitochondria isolated from human umbilical cord-derived mesenchymal stem cells were actively incorporated into neurons, resulting in improvement of sensorimotor functions [57]. Furthermore, mitochondria transplantation has shown a positive effect in ischemic and hemorrhagic stroke [58,59]. In addition, mitochondrial transplantation is being studied in cardiovascular diseases, inflammatory conditions, cancer, renal injury, and pulmonary damage.

### 6.2. Mitochondria Transplantation in Brain Disorders

Neurons use around 15% of the total amount of the body’s energy for their functions Therefore, mitochondria and the ATP they produce are extremely important for the proper functioning of neurons. Synapses are the primary consumers of ATP in the brain [60]. Because mitochondria play a crucial role in the functioning of brain neurons, that is why almost all brain pathologies are accompanied by mitochondrial abnormalities [61,62,63]. For example, mitochondrial damage has been shown to play an important role in brain damage caused by stroke [61]. Mitochondria dysfunction is also a common denominator in aging and brain-related diseases, such as depression, epilepsy, and neurodegenerative diseases (NDs) like Alzheimer’s (AD) and Parkinson’s disease (PD). Therefore, the research of MTT as a new treatment for different pathological conditions of the brain is a pathogenetically justified new treatment method, which has high hopes.

In some animal studies with artificially induced cerebral ischemia, intracerebral injection of mice allogeneic mitochondria extracted from liver cells [62] or intraarterial injection autologous mitochondria were used [63]. In comparison with control cortex, there were significantly less apoptotic and more proliferative oligodendrocyte progenitor cells (OPCs) in mitochondria-treated cortex. More importantly, higher levels of myelin basic protein (MBP) and more morphologically normal myelin-wrapped axons were observed in mitochondria-treated cortex at 21 days postinjury, as revealed by light and electron microscope [62]. Behavioral analysis showed better recovery of motor activity in mice treated with mitochondria.

Treatment with placental mitochondrial fractions immediately after reperfusion significantly decreased infarction after focal cerebral ischemia in mice [59]. The positive results obtained from the use of MTT in the treatment of ischemic stroke in animals indicate that mitochondrial transplantation is a potentially valuable method of treating ischemic stroke [62]. Norat et al. (2023) noted the elevated concentration of ATP in the stroked hemisphere, reduced infarct volume, and increased cell viability [63]. All of them have shown to reduce infarct size in treated animals compared to the untreated controls.

MTT improved survival and neurological recovery in post cardiac arrest rats. These results provide a foundation for future studies to promote the development of MTT as a novel therapeutic strategy to save lives currently lost after cardiac arrest [64].

The damaging effect of intracerebral hemorrhage (ICH) was reversed by intravenous transplantation of astrocytic mitochondria, which, upon entering the brain (and neurons), restored the mitochondrial enzyme manganese superoxide dismutase levels and reduced neurological deficits in male mice subjected to ICH [65].

C. Bamshad et al. (2023) indicate that human umbilical cord-derived mesenchymal stem cells-isolated mitochondrial transplantation improves motor function in a rat model of traumatic brain injury (TBI) via rescuing neuronal cells from apoptosis and alleviating astrogliosis and microglia activation, possibly as a result of restoring the lost mitochondrial content [58].

To treat the cerebellar ataxia of mice SJ Li et al. (2025) used liver-derived own mice mitochondria transplanted into the cerebellum. They saw improved mitochondrial function, reduced mitophagy, delayed apoptosis of Purkinje cells, and alleviated cerebellar ataxia up to 3 weeks. These findings demonstrate that mitochondria transplantation holds promise as a therapeutic approach for cerebellar degenerative diseases [66].

All the positive results obtained from the study of MTT in the treatment of various brain injuries indicate that transplanted mitochondria improve the course of the disease and improve the prognosis.

### 6.3. Mitochondria Transplantation in Heart Diseases

Myocardium has a high energy requirement: a healthy heart consumes from 6 to 30 kg of ATP daily. Mitochondria, accounting for 30% of the volume of cardiomyocytes, provide these high energy needs [52].

In heart disorders, MTT can significantly affect its functioning, promote the survival of cardiomyocytes, and reduce apoptosis by increasing energy synthesis, replacing damaged mtDNA and increasing cytokine levels. In this regard, the possibility of using MTT in the treatment of ischemic heart disease and during heart transplantation procedures is actively studied in animal models. Mitochondrial transplantation improved contractile function and respiratory capacity, reduced cellular apoptosis and oxidative stress in cardiomyocytes. Mitochondria isolated from various sources, including mouse hearts, mouse and human arterial blood, and human induced pluripotent stem cell-derived cardiomyocytes (hiPSC-CMs), all exerted similar cardioprotective effects [67].

D. Blitzer et al. (2019) demonstrated the efficacy of mitochondrial transplantation for the treatment of ischemia-reperfusion injury (IRI). Delayed MTT by intracoronary injection appreciably decreases myocardial infarct size, increasing regional and global myocardial function [68].

Z. Wu et al. (2024) reported successful mitochondrial transplantation by oral administration for ischemic heart disease (IHD) therapy. Results from animal models of IHD indicate that the accumulated mitochondria in the damaged heart tissue can regulate cardiac metabolism at the transcriptional level, thus preventing IHD progression. This strategy has the potential to change the therapeutic strategy used to treat IHD [69].

Similar results have been demonstrated in other studies of the effectiveness of MTT in heart disorders [70,71]. These results suggest that this may be a viable treatment modality in IRI, thus reducing long-term morbidity and mortality in cardiac surgical patients.

Currently, the study of MTT in acute kidney injuries [72,73], acute lung injuries [74], osteoarthritis [75], and in the treatment of liver injuries, including toxin- and ischemia-reperfusion injuries [76], continues.

### 6.4. Mitochondrial Transplantation in Humans

All the studies that we discussed earlier were conducted on animal models (mice, pigs, etc.). The first use of MTT in the treatment of humans was reported by S.M. Emani (2017) [77]. He and his team reported the first clinical trial of mitochondrial transplantation in Pediatric patients who required central extracorporeal membrane oxygenation (ECMO) support for ischemia-reperfusion-associated myocardial dysfunction after cardiac surgical procedure were eligible for mitochondrial autotransplantation. Mitochondrial harvest and isolation can be performed within 20 to 30 min during biopsy of nonischemic skeletal muscle. Of the five subjects, four demonstrated improvement in ventricular function and were successfully separated from ECMO support [77].

After 7 years, F. Baharvand et al. (2024) [78] conducted a prospective, triple-blinded, parallel-group, blocked randomized clinical trial to investigate the therapeutic effects and clinical outcomes of platelet-derived mitochondrial transplantation in 30 patients with acute ST-elevation myocardial infarction (STEMI). Fifteen subjects in the intervention group received autologous platelet-derived mitochondria through the intracoronary injection. Within 40 days of observation the intervention group had a slightly greater improvement in the left ventricular ejection fraction (LVEF) compared to the control group and experienced a significant enhancement in the exercise capacity (*p* < 0.001). These results appear to be beneficial and highly promising therapeutic option for patients of ischemic heart disease (IHD), but further in-depth studies with larger sample sizes along with longer follow-up periods are necessary for validating the clinical implications of these findings [78].

The next in-human clinical trial to assess the effectiveness and safety of mitochondria as a therapeutic intervention for idiopathic inflammatory myopathy (IIM). Mitochondria isolated from umbilical cord mesenchymal stem cells were designated as PN-101. The efficacy and safety of donated mitochondria was assessed using myoblasts derived from 9 adult patients with refractory polymyositis or dermatomyositis. The myoblasts derived from patients with IIM exhibited defects in mitochondrial function and myogenesis. Mitochondrial transplantation enhances muscle differentiation and mitochondrial function in IIM myoblasts, enhanced intracellular adenosine triphosphate content, cell viability, and myogenesis. In an in vivo model, donated mitochondria reduced myositis severity by exhibiting anti-inflammatory effects and restoring the C protein-induced myositis metabolic shift. Donated mitochondria demonstrated no severe adverse drug reactions and showed at least minimal improvement in the International Myositis Assessment and Clinical Studies Group -Total Improvement Scores compared with baseline [79].

However, despite the long history and wide coverage of these studies, the question of possible mechanisms of the therapeutic effect of “mitochondrial transplantation” remains open.

## 7. Conclusions

The problem of mitochondrial disorders is of great interest to scientists around the world. Unique methods have been proposed to address them, but not all of them have found wide clinical application at present. Mitochondrial replacement therapy remains controversial due to doubts about its safety and effectiveness. Many questions remain about how mitochondria from different donors can affect all cellular processes. The interaction between the mitochondrial and nuclear genomes may be disrupted, and in order to maintain a normal level of energy production in the cell, a connection between nuclear and mitochondrial DNA is needed, which can be disrupted in the presence of another person’s mitochondrial genome. Mitochondrial interventions have caused much debate, as changes in the genetic inheritance of born children remain untested and unstudied, and it is also unclear how these interventions will affect the health of future generations. In addition, a lot of controversy has arisen due to ethical considerations, since humanity first encountered children born from three parents. However, despite these doubts, objections, and disputes, these methods continue to be studied and applied in several countries, such as China, Spain, and Greece, allowing not only the development of science and technology, but also giving humanity hope for solving diseases associated with mitochondria [25,32,80,81].

MRT methods—despite the logical methodology and anticipated benefits—have shown that in biological systems, such as humans, no cellular technology model can guarantee a 100% positive result. This is because we currently lack a complete understanding of all cellular mechanisms. Therefore, studying the cell and the functioning of its structures is extremely relevant at present.

Regarding the ethical issues surrounding mitochondrial donation in MRT or MTT, which are under discussion, we believe that any donation is an attempt to help an unhealthy person. Typically, the issue of donation is only considered in extremely difficult clinical situations. Therefore, any donation should be evaluated based on clinical necessity. If donation can help avoid serious genetic problems (as in the case of MRT) or aid in the treatment of severe pathologies (such as myocardial ischemia or central nervous system pathology), then we believe that such a donation deserves ethical approval. In our opinion, it should also be remembered that we inherit mitochondria from our mother, and therefore children of the same mother have autologous mitochondria. This means that the mother and siblings could potentially be mitochondrial donors for one another.

## Figures and Tables

**Table 1:** Mitochondrial disorders.

Mitochondrial Disorder	Relative Frequency	Typical Feature(s)	Associated Feature(s)	Inheritance	Most Frequent Genetic Findings	Treatment of Choice
Alpers syndrome	Very rare	Childhood myocerebrohepatopathy		Autosomal recessive	*POLG* mutations with secondary mtDNA depletion	Symptomatic (avoid valproate)
Autosomal dominant optic atrophy (ADOA)	Rare	Optic neuropathy (blindness)		Autosomal dominant	*OPA1* mutations	Symptomatic
Coenzyme Q10 deficiency	Very rare	Ataxia or myopathy or multi-system disease		Autosomal recessive	Various nuclear genes	Coenzyme Q10
Kearns–Sayre syndrome (KSS)	Frequent	Ocular myopathy (ptosis, ophthalmoparesis)	Ataxia, cardiac conduction defects	Sporadic	Single large-scale deletion of mtDNA	Symptomatic
Leber hereditary optic neuropathy (LHON)	Very frequent	Optic neuropathy (blindness)		Maternal (low penetrance, higher in male smokers)	Various mtDNA mutations	Idebenone
Leigh syndrome	Frequent	Severe pediatric encephalopathy		Autosomal recessive, X-linked or maternal	Various nuclear or mtDNA mutations (e.g., m.8993T>G)	Symptomatic
Mitochondrial encephalopathy with lactic acidosis and stroke-like episodes (MELAS)	Frequent	Stroke-like episodes	Cardiac involvement, hearing loss, diabetes	Maternal	m.3243A>G	Symptomatic
Myoclonic encephalopathy with ragged-red fiber (MERRF)	Frequent	Myoclonus	Ataxia, myopathy	Maternal	m.8344A>G	Symptomatic (e.g., Levetiracetam)
Mitochondrial neurogastrointestinal encephalomyopathy (MNGIE)	Very rare	Gastrointestinal dysmotility	Leukodystrophy, ocular myopathy, peripheral neuropathy	Autosomal recessive	*TYMP* mutations	Liver transplantation
Neuropathy, ataxia, retinitis pigmentosa (NARP)	Rare	Ataxia	Neuropathy, retinitis pigmentosa	Maternal	m.8993T>G	Symptomatic
Non syndromic hearing loss (NSHL)	Frequent	Hearing loss		Maternal	m.1555A>G	Symptomatic (avoid aminoglycosides)
Progressive external ophthalmoplegia (PEO)	Very frequent	Ocular myopathy	Myopathy	Autosomal dominant, recessive, maternal, or sporadic	Various nuclear genes with secondary mtDNA multiple deletions, various mtDNA point mutations, and mtDNA single large-scale deletions	Symptomatic

## Abbreviations

The following abbreviations are used in this manuscript:

DNA	Deoxyribonucleic acid
mtDNA	Mitochondrial DNA
MRT	Mitochondrial Replacement Therapy
MTT	Mitochondrial Transplantation Therapy
PNT	Pronuclear transfer technique
MST	Maternal spindle transfer
PBT	Polar body genome transfer
GVT	Germinal vesicle transfer
AUGMENT	Autologous germline mitochondrial energy transfer
ICSI	Intracytoplasmic sperm injection
ATP	Adenosine triphosphate
AIF	Apoptosis-inducing factor
ROS	Reactive oxygen species
OS	Oxidative stress
CNS	Central nervous system
RNA	Ribonucleic acid
iPSCs	Induced pluripotent stem cells
FDA	Food and Drug Administration
IOM	Institute of Medicine
OSCs	Oogonial stem cells
IVF	In vitro fertilization
MT	Mitochondrial transfer
TNTs	Tunneling nanotubes
GJs	Gap junctions
EVs	Extracellular vesicles
MSCs	Mesenchymal stromal/stem cells
NDs	Neurodegenerative diseases
AD	Alzheimer’s disease
PD	Parkinson’s disease
OPCs	Oligodendrocyte progenitor cells
ICH	Intracerebral hemorrhage
TBI	Traumatic brain injury
hiPSC-CMs	Human induced pluripotent stem cell-derived cardiomyocytes
IRI	Ischemia-reperfusion injury
IHD	Ischemic heart disease
ECMO	Extracorporeal membrane oxygenation
STEMI	ST-elevation myocardial infarction
LVEF	Left ventricular ejection fraction
IIM	Idiopathic inflammatory myopathy

## References

[1] Holt I.J., Harding A.E., Morgan-Hughes J.A. (1988). Deletions of muscle mitochondrial DNA in patients with mitochondrial myopathies. Nature.

[2] Wallace D.C., Singh G., Lott M.T., Hodge J.A., Schurr T.G., Lezza A.M., Elsas L.J., Nikoskelainen E.K. (1988). Mitochondrial DNA mutation associated with Leber’s hereditary optic neuropathy. Science.

[3] Orsucci D., Caldarazzo Ienco E., Rossi A., Siciliano G., Mancuso M. (2021). Mitochondrial Syndromes Revisited. J. Clin. Med..

[4] Kang E., Wu J., Gutierrez N.M., Koski A., Tippner-Hedges R., Agaronyan K., Platero-Luengo A., Martinez-Redondo P., Ma H., Lee Y. (2016). Mitochondrial replacement in human oocytes carrying pathogenic mitochondrial DNA mutations. Nature.

[5] Tavare A. (2012). Scientists are to investigate “three parent IVF” for preventing mitochondrial diseases. BMJ.

[6] Pozdnyakova A.A., Volodina M.A., Rshtuni S.D., Marchenko L.A., Vysokih M.Y. (2015). Mitohondrial’naya disfunkciya kak odna iz vozmozhnyh prichinnarusheniya follikulo—I steroidogeneza pri prezhdevremennoj nedostatochnosti yaichnikov. Akusherstvo. Ginekol. Reprodukciya.

[7] Chen S., Li Q., Shi H., Li F., Duan Y., Guo Q. (2024). New insights into the role of mitochondrial dynamics in oxidative stress-induced diseases. Biomed. Pharmacother..

[8] Craven L., Tang M.X., Gorman G.S., De Sutter P., Heindryckx B. (2017). Novel reproductive technologies to prevent mitochondrial disease. Hum. Reprod. Update.

[9] Fragouli E., McCaffrey C., Ravichandran K., Spath K., Grifo J.A., Munné S., Wells D. (2017). Clinical implications of mitochondrial DNA quantification on pregnancy outcomes: A blinded prospective non-selection study. Hum. Reprod..

[10] Gu Y.Y., Zhao X.R., Zhang N., Yang Y., Yi Y., Shao Q.H., Liu M.X., Zhang X.L. (2024). Mitochondrial dysfunction as a therapeutic strategy for neurodegenerative diseases: Current insights and future directions. Ageing Res. Rev..

[11] Xu L., Shi R. (2016). Weigh and wait: The prospect of mitochondrial gene replacement. Hum. Fertil..

[12] DiMauro S. (2019). A Brief History of Mitochondrial Pathologies. Int. J. Mol. Sci..

[13] Rebolledo-Jaramillo B., Su M.S., Stoler N., McElhoe J.A., Dickins B., Blankenberg D., Korneliussen T.S., Chiaromonte F., Nielsen R., Holland M.M. (2014). Maternal age effect and severe germ-line bottleneck in the inheritance of human mitochondrial DNA. Proc. Natl. Acad. Sci. USA.

[14] Mitalipov S., Amato P., Parry S., Falk M.J. (2014). Limitations of preimplantation genetic diagnosis for mitochondrial DNA diseases. Cell Rep..

[15] Molnar T., Lehoczki A., Fekete M., Varnai R., Zavori L., Erdo-Bonyar S., Simon D., Berki T., Csecsei P., Ezer E. (2024). Mitochondrial dysfunction in long COVID: Mechanisms, consequences, and potential therapeutic approaches. Geroscience.

[16] Rebrikov D.V. (2016). Human Genome Editing.

[17] Bhai S., Hirano M. (2025). Diagnosis of Primary Mitochondrial Diseases. Muscle Nerve.

[18] Mancuso M., Papadopoulou M.T., Ng Y.S., Ardissone A., Bellusci M., Bertini E., Di Vito L., Evangelista T., Fons C., Hikmat O. (2024). Management of seizures in patients with primary mitochondrial diseases: Consensus statement from the InterERNs Mitochondrial Working Group. Eur. J. Neurol..

[19] Wen H., Deng H., Li B., Chen J., Zhu J., Zhang X., Yoshida S., Zhou Y. (2025). Mitochondrial diseases: From molecular mechanisms to therapeutic advances. Signal Transduct. Target. Ther..

[20] Selvanathan A., Teo J., Parayil Sankaran B. (2024). Hematologic Manifestations in Primary Mitochondrial Diseases. J. Pediatr. Hematol. Oncol..

[21] Heiduschka S., Prigione A. (2025). iPSC models of mitochondrial diseases. Neurobiol. Dis..

[22] Wolf D.P., Mitalipov N., Mitalipov S. (2015). Mitochondrial replacement therapy in reproductive medicine. Trends Mol. Med..

[23] Haimes E., Taylor K. (2017). Sharpening the cutting edge: Additional considerations for the UK debates on embryonic interventions for mitochondrial diseases. Life Sci. Soc. Policy..

[24] Sharma H., Singh D., Mahant A., Sohal S.K., Kesavan A.K. (2020). Samiksha Development of mitochondrial replacement therapy: A review. Heliyon.

[25] Subirá J., Soriano M.J., Del Castillo L.M., de Los Santos M.J. (2025). Mitochondrial replacement techniques to resolve mitochondrial dysfunction and ooplasmic deficiencies: Where are we now?. Hum. Reprod..

[26] Kaur S., Nagpal M. (2017). Recent advancement in human reproduction three-parent babies: A technique to neutralize mitochondrial disease load- A boon or a bane for society?. Curry Trends Diagn. Treat..

[27] Labarta E., Santos M.J.d.L., Escriba M.J., Pellicer A., Herriaz S. (2019). Mitochondria as a tool for oocyte rejuvenation. Fertil. Steril..

[28] Jose F., Lekshmi S., Lal S., Jjiju V., Abraham E. (2017). Three parent child: A review. Int. J. Nov. Trends Pharm. Sci..

[29] Schmerler S., Wessel G.M. (2011). Polar bodies—More a lack of understanding than a lack of respect. Mol. Reprod. Dev..

[30] Wang T., Sha H., Ji D., Zhang H.L., Chen D., Cao Y., Zhu J. (2014). Polar body genome transfer for preventing the transmission of inherited mitochondrial diseases. Cell.

[31] Neupane J., Vandewoestyne M., Ghimire S., Lu Y., Qian C., Van Coster R., Gerris J., DeRoo T., Deforce D., De Sutter P. (2014). Assessment of nuclear transfer techniques to prevent the transmission of heritable mitochondrial disorders without compromising embryonic development competence in mice. Mitochondrion.

[32] Sendra L., García-Mares A., Herrero M.J., Aliño S.F. (2021). Mitochondrial DNA Replacement Techniques to Prevent Human Mitochondrial Diseases. Int. J. Mol. Sci..

[33] Zaidi A.A., Makova K.D. (2019). lnvestigating mitonuclear interactions in human admixed populations. Nat. Ecol. Evol..

[34] https://www.fda.gov/vaccines-blood-biologics/cellular-gene-therapy-products/therapeutic-cloning-and-genome-modification.

[35] Zhang J. (2016). World’s First Baby Born from New Procedure Using DNA of Three People.

[36] Reardon S. (2017). Genetic details of controversial ‘three-parent baby’ revealed. Nature.

[37] Kumar A., Choudhary A., Munshi A. (2024). Epigenetic reprogramming of mtDNA and its etiology in mitochondrial diseases. J. Physiol. Biochem..

[38] Hamzelou J. (2016). ‘3-parent baby’ Success. New Sci..

[39] Woods D.C., Tilly J.L. (2015). Autologous Germline Mitochondrial Energy Transfer (AUGMENT) in Human Assisted Reproduction. Semin. Reprod. Med..

[40] Li L., Clevers H. (2010). Coexistence of quiescent and active adult stem cells in mammals. Science.

[41] Johnson J., Canning J., Kaneko T., Pru J.K., Tilly J.L. (2004). Germline stem cells and follicular renewal in the postnatal mammalian ovary. Nature.

[42] Zou K., Yuan Z., Yang Z., Luo H., Sun K., Zhou L., Xiang J., Shi L., Yu Q., Zhang Y. (2009). Production of offspring from a germline stem cell line derived from neonatal ovaries. Nat. Cell Biol..

[43] Grieve K.M., McLaughlin M., Dunlop C.E., Telfer E.E., Anderson R.A. (2015). The controversial existence and functional potential of oogonial stem cells. Maturitas.

[44] Silvestris E., D’Oronzo S., Cafforio P., DAmato G., Loverro G. (2015). Perspective in infertility: The ovarian stem cells. J. Ovarian Res..

[45] (2015). 31st Annual Meeting of the European Society of Human Reproduction and Embryology (ESHRE), Lisbon, Portugal, 14–17 July 2015.

[46] Labarta E., de Los Santos M.J., Herraiz S., Escribá M.J., Marzal A., Buigues A., Pellicer A. (2019). Autologous mitochondrial transfer as a complementary technique to intracytoplasmic sperm injection to improve embryo quality in patients undergoing in vitro fertilization—A randomized pilot study. Fertil. Steril..

[47] McCully J.D., Cowan D.B., Pacak C.A., Toumpoulis I.K., Dayalan H., Levitsky S. (2009). Injection of isolated mitochondria during early reperfusion for cardioprotection. Am. J. Physiol. Heart Circ. Physiol..

[48] Cozzolino M., Marin D., Sisti G. (2019). New frontiers in IVF: mtDNA and autologous germline mitochondrial energy transfer. Reprod. Biol. Endocrinol..

[49] Shanmughapriya S., Langford D., Natarajaseenivasan K. (2020). Inter and Intracellular mitochondrial trafficking in health and disease. Ageing Res. Rev..

[50] Masuzawa A., Black K.M., Pacak C.A., Ericsson M., Barnett R.J., Drumm C., Seth P., Bloch D.B., Levitsky S., Cowan D.B. (2013). Transplantation of autologously derived mitochondria protects the heart from ischemia-reperfusion injury. Am. J. Physiol. Heart Circ. Physiol..

[51] Iorio R., Petricca S., Mattei V., Delle Monache S. (2024). Horizontal mitochondrial transfer as a novel bioenergetic tool for mesenchymal stromal/stem cells: Molecular mechanisms and therapeutic potential in a variety of diseases. J. Transl. Med..

[52] Li X., Guan Y., Li C., Cheng H., Bai J., Zhao J., Wang Y., Peng J. (2025). Recent advances in mitochondrial transplantation to treat disease. Biomater. Transl..

[53] Ramirez-Barbieri G., Moskowitzova K., Shin B., Blitzer D., Orfany A., Guariento A., Iken K., Friehs I., Zurakowski D., Del Nido P.J. (2019). Alloreactivity and allorecognition of syngeneic and allogeneic mitochondria. Mitochondrion.

[54] Koch R.E., Josefson C.C., Hill G.E. (2017). Mitochondrial function, ornamentation, and immunocompetence. Biol. Rev. Camb. Philos. Soc..

[55] Desdín-Micó G., Soto-Heredero G., Aranda J.F., Oller J., Carrasco E., Gabandé-Rodríguez E., Blanco E.M., Alfranca A., Cussó L., Desco M. (2020). T cells with dysfunctional mitochondria induce multimorbidity and premature senescence. Science.

[56] Yu Z., Hou Y., Zhou W., Zhao Z., Liu Z., Fu A. (2021). The effect of mitochondrial transplantation therapy from different gender on inhibiting cell proliferation of malignant melanoma. Int. J. Biol. Sci..

[57] Nakano T., Irie K., Matsuo K., Mishima K., Nakamura Y. (2024). Molecular and cellular mechanisms of mitochondria transfer in models of central nervous system disease. J. Cereb. Blood Flow. Metab..

[58] Bamshad C., Habibi Roudkenar M., Abedinzade M., Yousefzadeh Chabok S., Pourmohammadi-Bejarpasi Z., Najafi-Ghalehlou N., Sato T., Tomita K., Jahanian-Najafabadi A., Feizkhah A. (2023). Human umbilical cord-derived mesenchymal stem cells-harvested mitochondrial transplantation improved motor function in TBI models through rescuing neuronal cells from apoptosis and alleviating astrogliosis and microglia activation. Int. Immunopharmacol..

[59] Nakamura Y., Lo E.H., Hayakawa K. (2020). Placental Mitochondria Therapy for Cerebral Ischemia-Reperfusion Injury in Mice. Stroke.

[60] Pekkurnaz G., Wang X. (2022). Mitochondrial heterogeneity and homeostasis through the lens of a neuron. Nat. Metab..

[61] Tian H., Chen X., Liao J., Yang T., Cheng S., Mei Z., Ge J. (2022). Mitochondrial quality control in stroke: From the mechanisms to therapeutic potentials. J. Cell. Mol. Med..

[62] Chen T., Zhu Y., Jia J., Meng H., Xu C., Xian P., Li Z., Tang Z., Wu Y., Liu Y. (2022). Mitochondrial Transplantation Promotes Remyelination and Long-Term Locomotion Recovery following Cerebral Ischemia. Mediat. Inflamm..

[63] Norat P., Sokolowski J.D., Gorick C.M., Soldozy S., Kumar J.S., Chae Y., Yagmurlu K., Nilak J., Sharifi K.A., Walker M. (2023). Intraarterial Transplantation of Mitochondria After Ischemic Stroke Reduces Cerebral Infarction. Stroke Vasc. Interv. Neurol..

[64] Hayashida K., Takegawa R., Endo Y., Yin T., Choudhary R.C., Aoki T., Nishikimi M., Murao A., Nakamura E., Shoaib M. (2023). Exogenous mitochondrial transplantation improves survival and neurological outcomes after resuscitation from cardiac arrest. BMC Med..

[65] Tashiro R., Bautista-Garrido J., Ozaki D., Sun G., Obertas L., Mobley A.S., Kim G.S., Aronowski J., Jung J.E. (2022). Transplantation of Astrocytic Mitochondria Modulates Neuronal Antioxidant Defense and Neuroplasticity and Promotes Functional Recovery after Intracerebral Hemorrhage. J. Neurosci..

[66] Li S.J., Zheng Q.W., Zheng J., Zhang J.B., Liu H., Tie J.J., Zhang K.L., Wu F.F., Li X.D., Zhang S. (2025). Mitochondria transplantation transiently rescues cerebellar neurodegeneration improving mitochondrial function and reducing mitophagy in mice. Nat. Commun..

[67] Sun X., Chen H., Gao R., Huang Y., Qu Y., Yang H., Wei X., Hu S., Zhang J., Wang P. (2023). Mitochondrial transplantation ameliorates doxorubicin-induced cardiac dysfunction via activating glutamine metabolism. iScience.

[68] Blitzer D., Guariento A., Doulamis I.P., Shin B., Moskowitzova K., Barbieri G.R., Orfany A., Del Nido P.J., McCully J.D. (2020). Delayed transplantation of autologous mitochondria for cardioprotection in a porcine model. Ann. Thorac. Surg..

[69] Wu Z., Chen L., Guo W., Wang J., Ni H., Liu J., Jiang W., Shen J., Mao C., Zhou M. (2024). Oral mitochondrial transplantation using nanomotors to treat ischaemic heart disease. Nat. Nanotechnol..

[70] Shin B., Saeed M.Y., Esch J.J., Guariento A., Blitzer D., Moskowitzova K., Ramirez-Barbieri G., Orfany A., Thedsanamoorthy J.K., Cowan D.B. (2019). A novel biological strategy for myocardial protection by intracoronary delivery of mitochondria:safety and efficacy. JACC Basic Transl. Sci..

[71] Mokhtari B., Delkhah M., Badalzadeh R., Ghaffari S. (2025). Mitochondrial transplantation combined with mitoquinone and melatonin: A survival strategy against myocardial reperfusion injury in aged rats. Exp. Physiol..

[72] Rossi A., Asthana A., Riganti C., Sedrakyan S., Byers L.N.B., Robertson J.V., Senger R.S., Montali F., Grange C., Dalmasso A. (2023). Mitochondria transplantation mitigates damage in an in vitro model of renal tubular injury and in an ex vivo model of DCD renal transplantation. Ann. Surg..

[73] Doulamis I.P., Guariento A., Duignan T., Kido T., Orfany A., Saeed M.Y., Weixler V.H., Blitzer D., Shin B., Snay E.R. (2020). Mitochondrial transplantation by intra-arterial injection for acute kidney injury. Am. J. Physiol. Renal Physiol..

[74] Pang Y.L., Fang S.Y., Cheng T.T., Huang C.C., Lin M.W., Lam C.F., Chen K.B. (2022). Viable allogeneic mitochondria transplantation improves gas exchange and alveolar-capillary permeability in rats with endotoxin-induced acute lung injuries. Int. J. Med. Sci..

[75] Zhong G., Liu W., Venkatesan J.K., Wang D., Madry H., Cucchiarini M. (2025). Autologous transplantation of mitochondria/rAAV IGF-I platforms in human osteoarthritic articular chondrocytes to treat osteoarthritis. Mol. Ther..

[76] Zhao Z., Hou Y., Zhou W., Keerthiga R., Fu A. (2021). Mitochondrial transplantation therapy inhibit carbon tetrachloride-induced liver injury through scavenging free radicals and protecting hepatocytes. Bioeng. Transl. Med..

[77] Emani S.M., Piekarski B.L., Harrild D., Del Nido P.J., McCully J.D. (2017). Autologous mitochondrial transplantation for dysfunction after ischemia-reperfusion injury. J. Thorac. Cardiovasc. Surg..

[78] Baharvand F., Habibi Roudkenar M., Pourmohammadi-Bejarpasi Z., Najafi-Ghalehlou N., Feizkhah A., Bashiri Aliabadi S., Salari A., Mohammadi Roushandeh A. (2024). Safety and efficacy of platelet-derived mitochondrial transplantation in ischaemic heart disease. Int. J. Cardiol..

[79] Kim J.Y., Kang Y.C., Kim M.J., Kim S.U., Kang H.R., Yeo J.S., Kim Y., Yu S.-H., Song B., Hwang J.W. (2025). Mitochondrial transplantation as a novel therapeutic approach in idiopathic inflammatory myopathy. Ann. Rheum. Dis..

[80] Fan X.Y., Guo L., Chen L.N., Yin S., Wen J., Li S., Ma J.Y., Jing T., Jiang M.X., Sun X.H. (2022). Reduction of mtDNA heteroplasmy in mitochondrial replacement therapy by inducing forced mitophagy. Nat. Biomed. Eng..

[81] Cohen I.G., Adashi E.Y., Gerke S., Palacios-González C., Ravitsky V. (2020). The Regulation of Mitochondrial Replacement Techniques Around the World. Annu. Rev. Genom. Hum. Genet..

